# Validity and reliability of the Dietary Sodium Restriction Questionnaire in peritoneal dialysis patients

**DOI:** 10.1371/journal.pone.0321177

**Published:** 2025-04-04

**Authors:** Yunyao Lin, Ruolin Li, Zhihao Chen, Yingxin Xie, Junyan Fang, Pu Li, Mingzi Chu, Yingli Liu

**Affiliations:** Department of Nephrology, Shanghai Ninth People’s Hospital, Shanghai Jiao Tong University School of Medicine, Shanghai, People’s Republic of China; Warren Alpert Medical School of Brown University: Brown University Warren Alpert Medical School, United States of America

## Abstract

**Objective:**

The Chinese version of Dietary Sodium Restriction Questionnaire (DSRQ) was adapted to evaluate its reliability and validity for measuring adherence to a sodium-restricted diet in peritoneal dialysis (PD) patients.

**Methods:**

Specific items related to peritoneal dialysis were added to create a PD version of the DSRQ (PD-DSRQ), which was administered to 135 patients undergoing PD. Item analysis was performed using the critical ratio and homogeneity tests. The reliability of the questionnaire was determined by assessing the internal consistency. Content validity was evaluated using the expert evaluation method, and construct validity was assessed via exploratory and confirmatory factor analyses.

**Results:**

The item analysis revealed correlation coefficients (R-values) ranging from 0.311 to 0.745 for each item, with statistically significant differences between the high and low subgroups for all items. The Cronbach’s α coefficients for the overall PD-DSRQ and the attitude, subjective norm, and perceived behavioral control subscales were 0.805, 0.892, 0.794, and 0.889, respectively. The item-level content validity index ranged from 0.83 to 1.00, and the scale-level content validity index/universal agreement was 0.9894. Exploratory factor analysis identified a three-factor structure consistent with the original DSRQ, except for Question 18. The three factors had eigenvalues of 5.302, 4.179, and 1.290, which explained 64.32% of the variance. The average variance extracted for each dimension was 0.5777, 0.5654, and 0.5259, and the composite reliability values were 0.8864, 0.7956, and 0.8802, respectively, demonstrating good convergent and discriminant validity.

**Conclusion:**

The PD-DSRQ encompasses general information and three dimensions: attitude, subjective norms, and perceived behavioral control. The questionnaire demonstrated strong reliability and validity, making it a reliable tool for assessing adherence to sodium-restricted diets in patients undergoing PD.

## 1 Introduction

Currently, more than 4 million people worldwide are expected to receive renal replacement therapy for end-stage kidney disease, of whom 78% are undergoing dialysis [1]. Approximately 11% of patients on dialysis undergo peritoneal dialysis (PD) [2]. PD has the advantages of simplicity of operation, better preservation of the residual renal function, and reduced hemodynamic impact [3]. Sodium ions are the main electrolytes of the extracellular fluid and regulate water, pH, and osmolality balance. Under normal conditions, the kidneys excrete most Na^ +^ ions. However, abnormal renal function in patients with ESKD, such as in those undergoing PD, leads to sodium retention, which is one of the most common electrolyte disorders. Cardiovascular events are closely related to volume overload caused by water-sodium retention and are the leading cause of death in patients undergoing PD [4]. Therefore, the water-sodium balance has an important influence on the adequacy of dialysis and prognosis of patients undergoing PD [5].

Sodium intake in adults should not exceed 2.3 g/d [6], and the global average dietary sodium intake for adults is nearly twice the recommended amount [7]. A 2018 study showed that the median dietary sodium intake of adult Chinese residents was 3.8 g/d, and 86.7% had an intake higher than 2 g/d [8]. Although the overall sodium intake of adult residents in China has decreased in recent years, it remains high. Patients undergoing PD also have a problem with sodium intake exceeding the recommended intake [9, 10], which is closely related to their elevated blood pressure [11, 12]. Moreover, sodium stored in the skin and other interstitial tissues increases with decreasing glomerular filtration rate, which may lead to abnormal changes in the structural function of the peritoneum [13]. A high-sodium diet may also lead to a rapid decline in residual renal function [14]. However, sodium intake is closely linked to overall food intake, and extremely low sodium intake has been associated with increased mortality risk [15]. Therefore, sodium intake in PD patients should be carefully managed to balance the benefits of restriction with potential risks, ultimately improving prognosis and quality of life.

Researchers often use 24-hour dietary recalls, 24-hour urine collections, and food frequency questionnaires (FFQ) to assess sodium intake, but they usually cannot accurately and conveniently measure sodium intake [16–18]. For PD patients, sodium intake is highly correlated with total sodium removal [19], which is the sum of dialytic and urinary sodium removal; however, discrepancies still exist due to misreporting of food portion sizes or composition. Currently, the development and introduction of assessment tools related to sodium-restricted diets for patients with PD are rare in China. Therefore, it is of great significance to develop an assessment tool for sodium-restricted diet compliance in patients with PD and analyze the factors influencing sodium-restricted diet for health management and improvement of prognosis.

## 2 Methods and materials

### 2.1 Research participants

Patients undergoing PD who had visited our hospital from January 2023 to June 2024 were selected as the study participants. The inclusion criteria were: stable PD for at least 3 months; over 18 years of age; and signing the informed consent form and able to truthfully fill in the questionnaire. The exclusion criteria were: patients who switch to hemodialysis treatment, receive renal transplantation, or die; poor general condition, serious condition, unable to eat on their own; concurrent malignant tumors or other serious somatic diseases; and psychiatric diseases, cognitive disorders, and other reasons for not being able to complete the questionnaire.

### 2.2 Research tools

Based on the Theory of Planned Behavior (TPB), Bentley et al. [20] developed the Dietary Sodium Restriction Questionnaire (DSRQ) to assess attitudes and behaviors toward sodium-restricted diet compliance in patients with heart failure. In 2018, Chen et al. [21] translated the DSRQ into Chinese for use with Chinese patients with heart failure, achieving good reliability and validity. The researchers obtained authorization to use and modify the Chinese version of the DSRQ. The content of the questionnaire was refined to adapt it for peritoneal dialysis, resulting in the Peritoneal Dialysis version of the DSRQ (PD-DSRQ) as shown in S1 File.

The modified questionnaire was still divided into two parts, but the basic information of patients was collected before the first part of the questionnaire, including patients’ sex, age, height, weight, marital status, education level, urine output, PD modality, ultrafiltration output, and underlying diseases, to understand the basic situation of the patients during the questionnaire’s use. The first part of the PD-DSRQ was increased from the original version’s 11 questions to 17. New questions (Question 5–7, 14–16) are about patients’ perception of the relevance of a low-sodium diet to ultrafiltration, edema, and blood pressure. Multiple-choice options were added for Question 2, 10 and 11. These changes were designed to facilitate the completion of the questionnaire by patients and to provide a more comprehensive description of the situation related to compliance with a low-salt diet in patients undergoing PD. The first part of the questionnaire was used only to describe and provide information about medical advice (or lack thereof) for a low-sodium diet, difficulties in following a low-sodium diet, and the extent to which patients felt that the diet was helpful in controlling their disease [20].

The second part, consistent with the original questionnaire, comprised three dimensions: attitude, subjective norms, and perceived behavioral control, with 16 items rated on a 5-point Likert scale. The attitude dimension refers to an individual’s beliefs about the outcomes of sodium-limiting behaviors and has six items. The subjective norms subscale refers to an individual’s subjective assessment of the social expectations and pressures of sodium-restricted dietary behaviors and has three items. The first two dimensions were positively scored on a scale of 1–5, ranging from “strongly disagree” to “strongly agree.” Perceived behavioral control refers to beliefs about the presence of facilitators or barriers to sodium-restricted diets, and comprised seven items, with scores ranging from 5 to 1 for “not at all” to “very much,” i.e., reverse scoring. Higher scores on the questionnaire indicated better attitudes and behaviors, that is, better compliance with the low-salt diet.

### 2.3 Research methods

The researchers used a unified guide to explain the purpose and significance of the study to the patients and informed them of the requirements for completing the questionnaire and the precautions to be taken. After obtaining patient consent, patients were instructed to complete the questionnaire, which was collected and checked immediately. During the survey, the patients were allowed to complete the questionnaire as much as possible without interference; for some patients who could not complete the questionnaire independently, the researchers asked the patients and their family members each question on the questionnaire in a question-and-answer mode and recorded it. The sample size was calculated to be 5–10 times the number of items on the scale. The PD-DSRQ has 16 items, and the proposed sample size is 80–160. In this study, 135 questionnaires were distributed and 135 valid questionnaires were collected from June 13 to August 20, 2024, with a valid recovery rate of 100%. This study was approved by the Institutional Ethics Committee (SH9H-2024-T214-1) and conducted in accordance with the Declaration of Helsinki. All participants provided written informed consent prior to inclusion in the study.

### 2.4 Statistical methods

The data were analyzed and tested using IBM SPSS Statistics 25.0 and IBM SPSS Amos 26.0 software. Count data was described by number of cases and percentage; normally distributed measures were described by mean ±  standard deviation, and non-normally distributed measures were described by median (first quartile, third quartile). The items were analyzed using item-total correlation and critical ratio methods. A correlation coefficient of r >  0.4 was considered to correlate the item with the total score, and a critical ratio of CR >  3 was considered to indicate that the item has discrimination [22]. Internal consistency was assessed to test the reliability of the questionnaire. A Cronbach’s α value greater than 0.70 was considered acceptable evidence of internal consistency [23]. The content validity was tested using the expert evaluation method. The content validity of the questionnaire was considered good if the item-level content validity index (I-CVI) was ≥ 0.78 and the scale-level content validity index (S-CVI) was > 0.90 [24]. A Kaiser-Meyer-Olkin (KMO) value > 0.7 and the P value of Bartlett’s spherical test statistic < 0.05 were considered good for factor analysis by principal component analysis [25, 26]. Exploratory and validation factor analyses were conducted to test the structural validity. A factor loading of > 0.4 for an entry indicates that the entry is representative of the corresponding factor [27]. Average variances extracted (AVE) >  0.5 and composite reliability (CR) >  0.7 for dimensions indicate good convergent and discriminant validity, respectively [28]. A P-value < 0.05 was considered to indicate statistical significance.

## 3 Results

### 3.1 General information

A total of 135 patients undergoing PD were included in this study: 79 (58.5%) were male, age 60.1 ± 13.5 years, height 166.0 ± 8.8 cm, weight 67.5 ± 13.3 kg, estimated glomerular filtration rate 4.6 ± 1.8 mL/min/1.73 m². A total of 112 (87.4%) patients were married and the remaining 17 were unmarried/divorced/widowed. There were 57 (42.2%) patients with anuria and 36 (26.7%) with oliguria. Ultrafiltration was 600 (200, 900) ml. The population included 122 (90.4%) patients undergoing Continuous Ambulatory PD (CAPD) and 13 (9.6%) patients undergoing Automated PD (APD). Systolic and diastolic blood pressure were 140.6 ± 15.7 and 80.6 ± 11.8 mmHg, respectively. A total of 124 patients had hypertension, 51 had diabetes mellitus, and 31 had other comorbidities, including coronary atherosclerotic heart disease, cerebral infarction, secondary hyperparathyroidism, gout/hyperuricemia, or other diseases.

### 3.2 Item analysis

The homogeneity of individual items on the overall scale was tested using Pearson’s correlation coefficient method. The higher the correlation coefficient, the better the homogeneity between the items and scale. The correlation coefficient R-value of each item with the total score of the PD-DSRQ ranged from 0.311 to 0.745 (all P < 0.001) (Table 1). The correlation coefficients of Items 19, 20, 21, and 23 were 0.377, 0.335, 0.319, and 0.311, respectively. Further linear regression analysis was performed for these items, and the value of adjusted R^2^ was 0.962, the Durbin-Watson value was 1.724, P < 0.001, and the variance inflation factor was < 5. This indicates relatively small autocorrelation and multicollinearity issues among these four items, good model stability, and significant effects on the total score of the DSRQ. Therefore, these items were retained for the analyses.

**Table 1:** Item analysis of the peritoneal dialysis version of the Dietary Sodium Restriction Questionnaire.

Item	Score (Mean ± SD)	Item-total Correlation (P value)	Critical ratio (P value)
**Q18**	4.20 ± 0.56	0.489(<0.001)	6.494(<0.001)
**Q19**	3.59 ± 0.92	0.377(<0.001)	4.329(<0.001)
**Q20**	3.51 ± 0.95	0.335(<0.001)	3.697(<0.001)
**Q21**	3.08 ± 1.01	0.319(<0.001)	2.649(0.010)
**Q22**	3.64 ± 0.90	0.423(<0.001)	4.462(<0.001)
**Q23**	3.24 ± 1.02	0.311(<0.001)	2.757(0.007)
**Attitude dimension**	21.26 ± 4.41		
**Q24**	4.19 ± 0.55	0.509(<0.001)	7.095(<0.001)
**Q25**	4.29 ± 0.53	0.519(<0.001)	6.879(<0.001)
**Q26**	4.05 ± 0.56	0.434(<0.001)	5.417(<0.001)
**Subjective norm dimension**	12.53 ± 1.39		
**Q27**	3.37 ± 1.18	0.509(<0.001)	8.004(<0.001)
**Q28**	2.96 ± 1.25	0.745(<0.001)	13.773(<0.001)
**Q29**	3.31 ± 1.32	0.579(<0.001)	8.205(<0.001)
**Q30**	3.14 ± 1.40	0.567(<0.001)	7.618(<0.001)
**Q31**	3.33 ± 1.18	0.521(<0.001)	5.999(<0.001)
**Q32**	2.68 ± 1.16	0.600(<0.001)	8.598(<0.001)
**Q33**	2.96 ± 1.15	0.611(<0.001)	8.566(<0.001)
**Perceived behavioral control dimension**	21.76 ± 6.75		
**Overall**	55.55 ± 7.87		

SD =  standard deviation

The critical ratio method was used to test the degree of discrimination for each item. The total scores of the PD-DSRQ of the 135 patients ranged from high to low, with samples scoring in the top 27% of the sample comprising the high subgroup and samples scoring in the bottom 27% comprising the low subgroup. For each item, we tested for a significant difference between the high and low groups to determine whether each item was discriminatory. The results of the independent samples t-test showed that the differences between the high and low groupings for each item were statistically significant (P < 0.05). Except for Questions 21 and 23, which had CR of 2.649 and 2.757, respectively, the CRs of the critical ratios for the remaining questions were all greater than three, as shown in Table 1.

### 3.3 Reliability analysis

Reliability was tested using the internal consistency of the three dimensions of the scale and the scale as a whole. The Cronbach’s α values of the attitude, subjective norms, perceived behavioral control dimensions and the whole scale were 0.892, 0.794, 0.889 and 0.805, respectively, which were all greater than 0.700. The Cronbach’s α values after deleting any item were all lower than 0.805 (Table 2).

**Table 2:** Reliability analysis of the peritoneal dialysis version of the Dietary Sodium Restriction Questionnaire.

Item	Cronbach’s α after deletion of terms	Cronbach’s α
**Q18**	0.771	
**Q19**	0.777	
**Q20**	0.781	
**Q21**	0.783	
**Q22**	0.774	
**Q23**	0.784	
**Attitude dimension**		0.892
**Q24**	0.770	
**Q25**	0.770	
**Q26**	0.773	
**Subjective norm dimension**		0.794
**Q27**	0.769	
**Q28**	0.742	
**Q29**	0.764	
**Q30**	0.767	
**Q31**	0.768	
**Q32**	0.760	
**Q33**	0.758	
**Perceived behavioral control dimension**		0.889
**Overall scale**		0.805

### 3.4 Validity analysis

In the expert evaluation method, the experts were asked to choose the relevance (or representativeness) of each item to the corresponding content dimension in the expert consultation questionnaire for content validity evaluation. The evaluation opinions were scored on a 4-level scale, irrelevant, weakly relevant, relevant, and very relevant, with scores ranging from 1 to 4. The content validity index was categorized into the content validity index at the item (I-CVI) and scale levels (S-CVI). The I-CVI was rated by a 6-member expert panel consisting of two nephrologists, two dietitians, a nurse, and a linguist, and all were in the range of 0.83 to 1.00 (>0.78); the S-CVI of universal agreement (S-CVI/UA) was 0.9375, and the mean S-CVI (S-CVI/ave) was 0.9894, i.e., the content validity of the scale was good. The Kendall W coefficient of concordance value was 0.465 (p < 0.001), indicating that expert opinions were statistically significant in agreeing with the applicability of the scale items.

**Fig 1:**
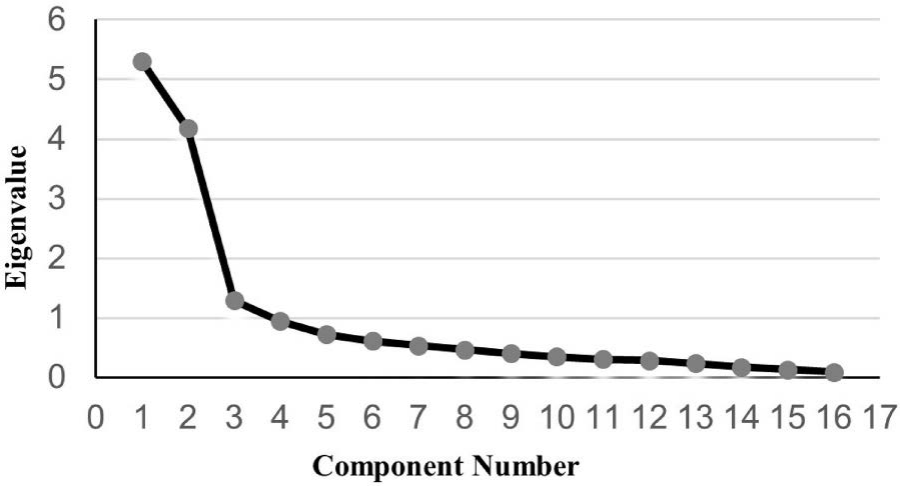
Scree plot of the peritoneal dialysis version of the Dietary Sodium Restriction Questionnaire. The Scree plot illustrates the eigenvalues of each factor. Three factors have eigenvalues greater than 1, which account for a significant portion of the total variance.

Before exploratory factor analysis, a statistical KMO test value of 0.842 and Bartlett’s spherical test statistic of 1410.281 (P < 0.001) indicated suitability for factor analysis. Structural validity was tested using a principal component analysis. The scree plot showed a three-factor structure (Fig 1). The results showed three factors with eigenvalues greater than 1 for the 16 items, and the eigenvalues of the three males were 5.302, 4.179, and 1.290, contributing 26.389%, 25.212%, and 15.727%, respectively, with a cumulative variance contribution of 67.320%. The loadings of each item on a single dimension were higher than 0.5, and all were valid. After varimax rotation, Questions 19–23, 24–26, and 27–33 belonged to the same dimensions as the original questionnaire. Question 18 was classified under the subjective norm dimension (Table 3).

**Table 3:** Three-factor structure of the peritoneal dialysis version of the Dietary Sodium Restriction Questionnaire.

Item	Dimension 1: Attitude	Dimension 2: Subjective norm	Dimension 3: Perceived behavioral control
**Q18**		0.533	
**Q19**	0.839		
**Q20**	0.855		
**Q21**	0.849		
**Q22**	0.703		
**Q23**	0.828		
**Q24**		0.769	
**Q25**		0.828	
**Q26**		0.794	
**Q27**			0.668
**Q28**			0.833
**Q29**			0.808
**Q30**			0.822
**Q31**			0.704
**Q32**			0.702
**Q33**			0.788

The factor analysis revealed three main factors, which correspond to the three dimensions of the Theory of Planned Behavior, with loadings greater than 0.5 for all items.

**Fig 2:**
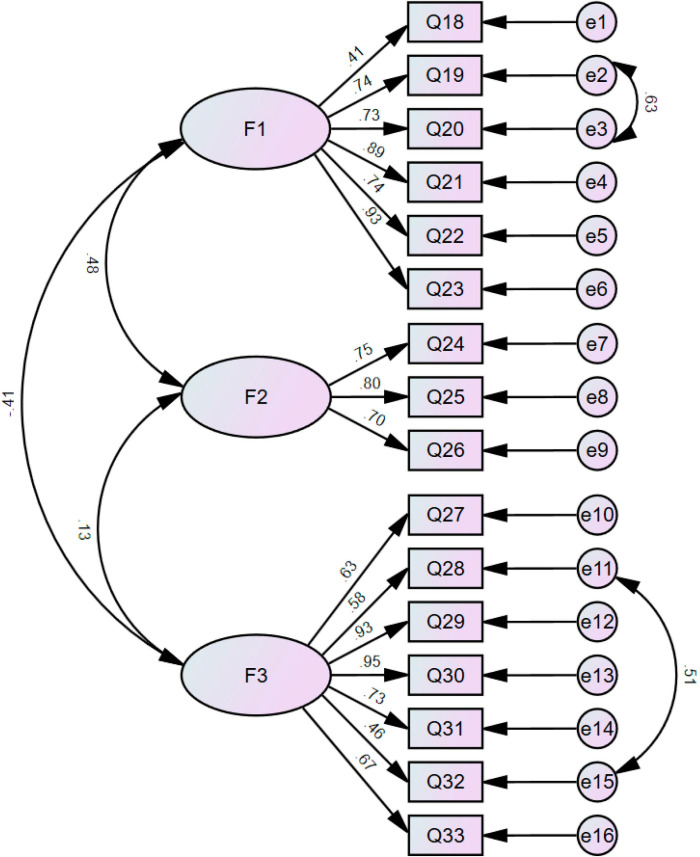
Structural equation model of the peritoneal dialysis version of the Dietary Sodium Restriction Questionnaire.

Because the dimensions of the scale were known, the model fit was tested using confirmatory factor analysis, as shown in Fig 2. Transferring Question 18, “It is important for me to follow a low-salt diet” to the subjective norms dimension showed no significant change in the fit indices when compared with maintaining the original dimensional structure. Considering that this question reflects patients’ awareness of the importance of a low-sodium diet and their attitudes toward a low-sodium diet, and that the loading for Question 18 in the attitude dimension was acceptable, the question was classified in the attitude dimension according to TPB. Examination of the model fit revealed that Question 19, “A low-salt diet prevents the increase of fluid in the body” and Question 20, “A low-salt diet will make my edema go down,” Question 28, “I do not like the taste of low-salt foods” and Question 32, “I like foods that are not low-salt” had large model chi-square values and insufficiently independent residuals. Considering the overlapping meanings of the questions, correlations between error variables e2 and e3, and between e11 and e15 were added to create a modified structural equation model, and the fit indices improved. The factor loadings of the latent variables of attitude, subjective norms, and perceived behavioral control corresponding to each item were all greater than 0.4, indicating that the latent variables for each item were representative. The convergent validity analysis suggested that the AVE values of the three dimensions were 0.5777, 0.5654, and 0.5259 (>0.5), and the CR values were 0.8864, 0.7956, and 0.8802 (>0.7), respectively, suggesting good convergent validity among the dimensions. The AVE value of each factor was greater than the correlation coefficient between that factor and other factors, indicating good discriminant validity.

## 4 Discussion

In this study, the average total score of the PD-DSRQ for the 135 respondents was 55.55, and the average scores for the 16 items ranged from 2.68 to 4.29. The lowest mean score was for Question 28 “I do not like the taste of low-salt foods” and the highest was for Question 18 “Following a low-salt diet is important to me.” Differences in the DSRQ scores between versions in different studies may be due to differences in patient populations, national cultural backgrounds, healthcare delivery systems, and the capacity and level of primary healthcare services. For example, the research institute has professional nutritionists who provide low-salt diet education to all patients undergoing PD, whereas Chinese residents are more accustomed to consuming foods cooked with various salty seasonings.

The total item correlation coefficients and item-total correlation analyses showed that all items of the PD-DSRQ were useful in understanding adherence to a low-sodium diet in patients undergoing PD. The scale would have been more reliable if the test items had a greater discriminatory power. Significant differences were observed in all items and overall scores between the high and low subgroups, suggesting that the PD-DSRQ can differentiate between patients’ sodium-restricted diet adherence based on the score level. In the item analysis, items 19, 20, 21, and 23 had correlation coefficients below 0.4 with the total score of the questionnaire, and the critical ratios of items 21 and 23 were < 3. However, these four items performed well in the linear regression analysis and reflected the attitudes of patients undergoing PD regarding the relationship between a low-salt diet and related symptoms; therefore, they were retained.

The calculation of Cronbach’s α coefficients may be prone to bias when small samples or scale score intervals are too close together [23]. Although the sample size of this study was smaller than that of the original study (n = 174), the Cronbach’s α values for all dimensions were higher than those of the original scale (0.88, 0.62, and 0.76) [20]. These results suggest that the dimensions and scale, in general, have good internal consistency and can assess the behavioral intentions of patients on a low-sodium diet.

The content validity of the PD-DSRQ was examined by the expert evaluation method, and the content validity indices at both the item and scale levels were acceptable, indicating good content validity. The experts agreed that the scale items can reflect the adherence to sodium-restricted diets of patients undergoing PD. Exploratory factor analysis validated the three-dimensional structure of the PD-DSRQ and explained 67.320% of the variance, similar to the Chinese version of the DSRQ for patients with heart failure (68.23%) [21]. Compared with the original study (54.2%) [20], the PD-DSRQ had a higher total explained variance and a stronger factor structure, which may be attributed to the fact that the content of this scale responded more closely to factors influencing sodium-restricted diet adherence in Chinese patients. Unlike in the present study, attitudes and subjective norms had a greater influence on low-sodium dietary behaviors. Question 18 was classified under the subjective norm dimension, which may reflect differences in the cultural, demographic, or clinical characteristics of the population. The differences in ethnicity (100% Chinese vs. 83.8% Caucasian), partner (87.4% vs. 61.9%), and mean years of education (17.8 vs. 11.8 years) in this study compared with the original study [20] may have influenced the composition of patients’ intentions, which were more influenced by attitudes and social expectations. In this study, we conducted the first validation factor analysis of the Chinese version of the DSRQ, which further validated that the three-dimensional structure of attitudes, subjective norms, and perceived behavioral control is consistent with the structure of the TPB and that it has good fitting ability, convergent validity, and discriminant validity.

Sodium restriction is an effective means of controlling blood pressure and improving edema for patients undergoing PD [29], and adherence to dietary sodium restriction remains an important goal. However, limiting the sodium intake can be challenging. Various tools have been developed to understand sodium intake behavior. The World Health Organization recommends 24-hour urine collection as the gold standard for assessing sodium intake; however, this method is time-consuming, labor-intensive, and difficult to roll out on a large scale [30]. Food records, such as 24-hour dietary recalls, food diaries, or food frequency questionnaires, are highly influenced by local eating habits and also suffer from low accuracy and sensitivity [18,31]. Few tools exist that consider patients’ behavioral intentions and compliance. Considering these general limitations, some researchers have turned to studying the assessment of sodium restriction adherence [32,33]. Therefore, it may not be accurate to focus solely on measuring health knowledge related to sodium-restricted diets to assess or increase adherence [34]. Simply informing patients about the importance of a low-sodium diet through health promotion does not necessarily lead to improved behavior or adherence. Therefore, it is important to investigate other factors that may lead to changes in intentions or actual behavior.

Common health behavior models include the theories of Knowledge, Attitude, and Practice (KAP) and the Theory of Planned Behavior (TPB). The KAP focuses on the effect of knowledge on behavior, whereas the TPB focuses on belief changes to predict behavioral intention. Researchers have developed a scale for patients undergoing PD based on KAP [35], which works well in the target domain. The TPB is commonly used to explain behavioral change, and it is believed that behavioral intention is the most important factor influencing behavior; behavioral intention is determined by attitudes, subjective norms, and perceived behavioral control [36]. The DSRQ was developed based on TPB, a widely developed and accepted theory for evaluating health-related behaviors [20]. After the emergence of the DSRQ, d’Almeida et al. [37], Korkmaz et al. [38], and Chen et al. [21] translated it into Portuguese, Turkish, and Chinese for patients with heart failure in various countries, achieving good reliability and validity. The DSRQ has been used and promoted to a certain extent abroad; Rodrigues et al. [39] and Wicaksana et al. [32] used the DSRQ in patients with hypertension, and Zhang et al. [40] used the DSRQ in patients with chronic kidney disease; they concluded that the DSRQ can be used as a tool to assess the cognition, attitudes, and behaviors of sodium-restricted diets in various types of patients.

The DSRQ is a reliable tool for understanding individual sodium restriction adherence, and can help researchers and clinicians assess patient adherence conveniently and quickly [20]. Personal beliefs regarding sodium-restricted diets were integrated into the DSRQ subscales of attitudes, subjective norms, and perceived behavioral control. Attitude refers to the PD patients’ positive or negative evaluations of a low-salt diet. Patients’ attitudes toward a low-salt diet directly influence their adoption of such a diet. Subjective norms refer to the social pressure that patients feel from significant others or groups; that is, whether others believe that the patient should follow a low-salt diet. Family, friends, medical professionals’ advice, and social trends can influence individuals’ health behaviors. Perceived behavioral control refers to the patients’ perception of their ability to adhere to a low-salt diet. If a person believes that a low-salt diet can bring positive results (such as improved edema), feels that the people around them are focused on a low-salt diet, and believes that they have the ability to stick to it (high perceived behavioral control), their behavioral intention will be stronger, making them more likely to adopt such behaviors. Exploring the subjectivity associated with sodium-restricted diets in patients undergoing PD can help professionals identify the difficulties and barriers encountered by patients. This study validated the PD-DSRQ as a reliable tool that can provide information about sodium-restricted dietary adherence in patients undergoing PD and can provide points for poor adherence to adequately plan and implement individual interventions.

The study assessed the reliability and validity of the PD-DSRQ in evaluating sodium-restricted dietary adherence among patients undergoing PD. To further verify the clinical practicality and credibility of this questionnaire, our follow-up research will explore the correlation between patients’ sodium intake and questionnaire scores. Obtaining comprehensive and accurate daily sodium intake data poses significant challenges due to the diversity of patients’ diets, the complexity of the record, and adherence issues. Sodium removal in PD patients is closely related to sodium intake and is a commonly used indicator for estimating sodium intake, with higher clinical feasibility than complex dietary records. Therefore, our subsequent study employs total sodium clearance to estimate sodium intake, facilitating a more comprehensive evaluation of the questionnaire’s usability. Our preliminary analysis shows a significant correlation between questionnaire scores and total sodium removal suggesting that the questionnaire reflects patients’ sodium intake. Future studies will expand the sample size and further investigate the applicability of PD-DSRQ.

In this study, the PD-DSRQ was applied only to single-center patients undergoing PD, while it is important to note that dietary habits and PD regimen compositions can vary across different populations and regions. Different backgrounds, cultures, and food availability may be reflected in individual behavioral intentions, which in turn influence sodium intake behavior [41,42]. In other cultures, attitudes, subjective norms, and perceived behavioral control may differ from those of the population in this study. Therefore, the PD-DSRQ needs to be further validated in patients undergoing PD in other regions and countries, which, in turn, will provide a scientific basis for physicians to evaluate adherence to sodium-restricted diets. In addition, to rigorously validate that the PD-DSRQ can assess patients’ sodium intake behavior, its correlation with actual sodium intake needs to be definitively verified.

## 5 Conclusion

The peritoneal dialysis version of the Dietary Sodium Restriction Questionnaire (PD-DSRQ) is a valid and reliable tool for assessing attitudes, subjective norms, and perceived behavioral control of PD patients on a low-sodium diet and for understanding facilitators and barriers to sodium restriction adherence. Professionals can use the PD-DSRQ to investigate and assess the actual behavior of low-sodium diet and long-term changes in patients undergoing PD, which can help provide appropriate recommendations for behavior modification, reduce water and sodium retention, and ultimately improve patients’ quality of life and prognosis.

## Supporting information

S1 File The peritoneal dialysis version of the Dietary Sodium Restriction Questionnaire. (DOCX)

S2 File Data. (XLSX)
