# Supplementary material for: Validity and reliability of the Dietary Sodium Restriction Questionnaire in peritoneal dialysis patients
Source: PLoS One. 2025 Apr 4;20(4):e0321177. doi: 10.1371/journal.pone.0321177 (PMC11970638; doi:10.1371/journal.pone.0321177)
Supplement: S1 File — (DOCX) [file pone.0321177.s001.docx]

**The peritoneal dialysis version of Dietary Sodium Restriction Questionnaire**

Water-sodium balance is considered a key factor influencing dialysis adequacy and patient prognosis. Sodium is one of the main determinants of extracellular fluid, and sodium restriction can help control both extracellular fluid and blood pressure. If sodium retention leads to volume overload, the likelihood of cardiovascular events increases. Cardiovascular events are the leading cause of death among peritoneal dialysis patients. Therefore, monitoring sodium intake in peritoneal dialysis patients is crucial for improving prognosis and other related outcomes.

- Please fill in your basic information：

| Age (years) |  | Gender | ○Male ○ Female |
| --- | --- | --- | --- |
| Height (cm) |  | Weight (kg) |  |
| Marital status | ○ Single ○ Married ○ Divorced ○ Widowed | | |
| Educational level | ○ Illiterate/Semi-literate ○ Primary school ○ Junior high school  ○ High school/Vocational school ○ College and above | | |

- [Single-choice question] What is your average daily urine output recently?

○ No urine ○ Less than 100ml per day ○ 100-400ml per day ○ More than 400ml per day

- [Fill-in question] What is your PD modality? ____;

What is your average peritoneal dialysis ultrafiltration volume per day recently? ____ ml.

- [Fill-in question] What is your recent morning systolic blood pressure? ______ mmHg; diastolic blood pressure? ______ mmHg.
- [Multiple-choice question] Besides chronic kidney disease, please select any other diagnosed conditions:

□ No other conditions □ Diabetes □ Hypertension □ Other ______________

**Section I**

1. Were you prescribed a low-salt diet by your health care provider?

○ Yes; if yes, continue with question 2

○ No; if no, skip to question 9

2. **[Multiple choice]** What specific instructions were you given?

*□ Consume less than 3 grams of salt daily □ 2 grams of sodium diet daily*

*□ Reduce salt intake □ Eat less of certain foods*

*□ Other _________________*

3. How closely do you follow your prescribed low-salt diet?

○ never ○ sometimes ○ most times ○ always

4. How easy is it to follow your prescribed low-salt diet?

○ very hard ○ hard ○ easy ○ very easy

*5. Has following this diet helped you manage your peritoneal dialysis ultrafiltration?*

○ not at all ○ slightly ○ a lot

*6. Has following this diet helped you manage edema?*

○ not at all ○ slightly ○ a lot

*7. Has following this diet helped you manage your blood pressure?*

○ not at all ○ slightly ○ a lot

8. Has following this diet helped you manage your heart condition?

○ not at all ○ slightly ○ a lot

Some people choose to follow a low-salt diet even though they were never told to do so by a health care provider.

9. Do you try to follow a low-salt diet?

○ Yes; if yes, continue with question 10

○ No; if no, please skip to question 18

10. **[Multiple choice]** Why did you decide to follow this diet?

*□ Read about it in newspapers or books □ Heard about it on news programs*

*□ Saw it on your phone □ Recommended by family or friends □ Other ________________*

11. **[Multiple choice]** What specifically do you do?

*□ Use less salt, soy sauce, and other seasonings while cooking*

*□ Monitor sodium content in food, and eat less sodium-rich foods*

*□ Reduce dining out or choose low-sodium foods when eating out*

*□ Other _________________*

12. How closely do you follow this diet?

○ not at all ○ slightly ○ a lot

13. How easy is it to follow your prescribed low-salt diet?

○ not at all ○ slightly ○ a lot

*14. Has following this diet helped you manage your peritoneal dialysis ultrafiltration??*

○ not at all ○ slightly ○ a lot

*15. Has following this diet helped you manage edema?*

○ not at all ○ slightly ○ a lot

*16. Has following this diet helped you manage your blood pressure?*

○ not at all ○ slightly ○ a lot

17. Has following this diet helped you manage your heart condition?

○ not at all ○ slightly ○ a lot

**Section II**

**Attitude Subscale**

| For each of the statements below, indicate how much you agree with the statement by circling the appropriate number using the scale to the right. | Strongly Disagree |  |  |  | Strongly Agree |
| --- | --- | --- | --- | --- | --- |
| 12. It is important for me to follow my low-salt diet. | 1 | 2 | 3 | 4 | 5 |
| 13. Eating a low-salt diet will keep fluid from building up in my body. | 1 | 2 | 3 | 4 | 5 |
| 14. Eating a low-salt diet will keep my swelling down. | 1 | 2 | 3 | 4 | 5 |
| 15. Eating a low-salt diet will help me breathe easier. | 1 | 2 | 3 | 4 | 5 |
| 16. When I follow a low-salt diet, I feel better. | 1 | 2 | 3 | 4 | 5 |
| 17. Eating a low-salt diet will keep my heart healthy. | 1 | 2 | 3 | 4 | 5 |

**Subjective Norm Subscale**

| 18. My spouse or other family members think l should follow a low-salt dict. | 1 | 2 | 3 | 4 | 5 |
| --- | --- | --- | --- | --- | --- |
| 19. Generally, I want to do what my doctor thinks I should do. | 1 | 2 | 3 | 4 | 5 |
| 20. Generally, I want to do what my spouse or family members think I should do. | 1 | 2 | 3 | 4 | 5 |

**Perceived Behavioral Control Subscale**

| Indicate below how much the following items keep you from following a low-salt diet by circling the appropriate number using the scale to the right. | Not at all |  |  |  | A lot |
| --- | --- | --- | --- | --- | --- |
| 21. Don't understand or know how. | 1 | 2 | 3 | 4 | 5 |
| 22. Taste of low-salt foods. | 1 | 2 | 3 | 4 | 5 |
| 23. Can't pick out low-salt foods in restaurants. | 1 | 2 | 3 | 4 | 5 |
| 24. The restaurants I like don't serve low-salt foods. | 1 | 2 | 3 | 4 | 5 |
| 25. Can't pick out low-salt foods at the grocery. | 1 | 2 | 3 | 4 | 5 |
| 26. The foods I like to eat are not low-salt. | 1 | 2 | 3 | 4 | 5 |
| 27. I don't have the willpower to change my diet. | 1 | 2 | 3 | 4 | 5 |

【腹透患者限钠饮食问卷】

水钠平衡被认为是影响透析充分性及患者预后的主要因素。钠是细胞外液的主要决定因素之一，限制钠可控制细胞外液和血压。若钠潴留引起容量超负荷，心血管事件发生的可能也随之增高，心血管事件是造成腹透患者死亡最重要的原因。因此，关注腹透患者的钠摄入，对于改善预后等具有重要意义。

- 请填写您的基本信息：

| 年龄（岁） |  | 性别 | ○男 ○女 |
| --- | --- | --- | --- |
| 身高（cm） |  | 体重（kg） |  |
| 婚姻状况 | ○未婚 ○已婚 ○离异 ○丧偶 | | |
| 文化程度 | ○文盲、半文盲 ○小学 ○初中 ○高中、技校 ○大专及以上 | | |

- [单选题]最近您每日平均尿量为：

○无尿 ○每日小于100ml ○每日100~400ml ○每日大于400ml

- [填空题]您的腹膜透析模式是：__________；最近您一日平均腹透超滤量为：__________ml。
- [填空题]最近您的清晨收缩压：________mmHg；舒张压：________mmHg。
- [多选题]除慢性肾脏病外，请选择您已确诊的其他疾病：

□无其他疾病 □糖尿病 □高血压 □其他 _________________

第一部分

1、你的医生给你开过低盐饮食或曾建议你低盐饮食吗？ [单选题]

○是的 **(请继续第2题)**

○没有 **(请跳至第9题)**

2、你得到了什么具体的指示? [多选题]

□每日食用小于3克的盐 □每日2克钠饮食

□减少盐摄入 □少吃某食品 □其他 _________________

3、你是否严格遵守你的低盐食谱？ [单选题]

○从不 ○有时 ○大多数时候 ○总是

4、坚持规定的低盐饮食容易吗? [单选题]

○非常困难 ○困难 ○容易 ○非常容易

*5、遵循低钠饮食与你腹透超滤多少有关系吗? [单选题]*

○完全没有关系 ○有一点关系 ○非常有关系

*6、遵循这种饮食对你控制水肿有帮助吗? [单选题]*

○完全没有帮助 ○有一点帮助 ○非常有帮助

*7、遵循这种饮食对你控制血压有帮助吗? [单选题]*

○完全没有帮助 ○有一点帮助 ○非常有帮助

8、遵循这种饮食对你控制心脏状况有帮助吗? [单选题]

○完全没有帮助 ○有一点帮助 ○非常有帮助

**（填写第8题后，请跳至第18题）**

9、你是否曾试着遵循低盐饮食? [单选题]

○有过 **(请继续第10题)**

○没有 **(请跳至第18题)**

10、你为什么决定遵循低钠或低盐饮食? [多选题]

*□在报纸书籍上读到 □在新闻节目上听说*

*□在手机上看到 □家人朋友推荐 □其他 _________________*

11、为了低盐饮食，你做了什么？ [多选题]

*□做菜时少加盐、酱油等调味品 □关注食品含钠量，少吃含钠多的食品*

*□减少外出就餐次数，或在外尽量选择低钠食品 □其他 _________________*

12、你是否严格遵守你的低盐食谱？ [单选题]

○从不 ○有时 ○大多数时候 ○总是

13、坚持规定的低盐饮食容易吗? [单选题]

○非常困难 ○困难 ○容易 ○非常容易

*14、遵循低钠饮食与你腹透超滤多少有关系吗? [单选题]*

○完全没有关系 ○有一点关系 ○非常有关系

*15、遵循这种饮食对你控制水肿有帮助吗? [单选题]*

○完全没有帮助 ○有一点帮助 ○非常有帮助

*16、遵循这种饮食对你控制血压有帮助吗? [单选题]*

○完全没有帮助 ○有一点帮助 ○非常有帮助

17、遵循这种饮食对你控制心脏状况有帮助吗? [单选题]

○完全没有帮助 ○有一点帮助 ○非常有帮助

第二部分

- **请选择您对每个陈述的同意程度。**

|  | 强烈反对 | 反对 | 不一定 | 同意 | 强烈同意 |
| --- | --- | --- | --- | --- | --- |
| 18.遵循低盐饮食对我重要。 | ○ | ○ | ○ | ○ | ○ |
| 19.吃低盐饮食可以避免身体中液体的增加。 | ○ | ○ | ○ | ○ | ○ |
| 20.吃低盐饮食可以让我的水肿消退。 | ○ | ○ | ○ | ○ | ○ |
| 21.吃低盐饮食可以让我的呼吸更加轻松。 | ○ | ○ | ○ | ○ | ○ |
| 22.当遵循低盐饮食时，我感觉更好。 | ○ | ○ | ○ | ○ | ○ |
| 23.吃低盐饮食会让我的心脏健康。 | ○ | ○ | ○ | ○ | ○ |

- **请选择您对每个陈述的同意程度。**

|  | 强烈反对 | 反对 | 不一定 | 同意 | 强烈同意 |
| --- | --- | --- | --- | --- | --- |
| 24.我的配偶和其他家庭成员认为我应该遵循低盐饮食 | ○ | ○ | ○ | ○ | ○ |
| 25.一般来说，我想做我的医生认为我应该做的事。 | ○ | ○ | ○ | ○ | ○ |
| 26.一般来说，我想做我的配偶和家人认为我应该做的事。 | ○ | ○ | ○ | ○ | ○ |

- **请指出下列项目对您坚持低盐饮食的影响程度。**

|  | 完全不影响 | 不太影响 | 不一定 | 有点影响 | 影响很大 |
| --- | --- | --- | --- | --- | --- |
| 27.不明白或不知道如何执行低盐饮食的要求。 | ○ | ○ | ○ | ○ | ○ |
| 28.低盐饮食的味道不好。 | ○ | ○ | ○ | ○ | ○ |
| 29.在餐馆就餐时不会挑选低盐食品。 | ○ | ○ | ○ | ○ | ○ |
| 30.我喜欢的餐馆不供应低盐食品。 | ○ | ○ | ○ | ○ | ○ |
| 31.在食品店里不会挑选低盐食品。 | ○ | ○ | ○ | ○ | ○ |
| 32.我喜欢吃的食物不是低盐的。 | ○ | ○ | ○ | ○ | ○ |
| 33.我没有毅力去改变我的饮食。 | ○ | ○ | ○ | ○ | ○ |

【问卷结束，感谢您的参与！】
